# Implementing of a problem-based learning strategy in a Saudi medical school: requisites and challenges

**DOI:** 10.5116/ijme.5aae.2db8

**Published:** 2018-03-27

**Authors:** Mutasim E. Ibrahim, Abdullah M. Al-Shahrani

**Affiliations:** 1 Department of Medical Microbiology, College of Medicine, University of Bisha, Saudi Arabia; 2 Department of Family Medicine, College of Medicine, University of Bisha, Saudi Arabia

**Keywords:** Problem-based learning, strategy, Saudi medical school, requisites and challenges, Saudi Arabia

## Introduction

Due to the current changes in healthcare realities and the continuing expansion of knowledge, there is an increasing demand for cognitive and problem-solving abilities to recognize patient and society’s needs.^1^^,^^2^ The health needs might be accomplished by making changes in traditional learning strategies to a more student-centered approach and utilizing flexible educational strategies as well as problem-based learning (PBL).^2^ The educational goals of PBL allow students to integrate basic science knowledge into the solution of clinical problems and ultimately serve better the future patient.^3^^,^^4^ In this article, we explore our experience in implementation of a hybrid PBL curriculum at the University of Bisha, College of Medicine (UBCOM), Saudi Arabia. Initially, we will briefly summarize the existing state of medical education in Saudi medical schools, followed by a description of PBL curriculum structure and its implementation challenges faced at UBCOM.

### Current status of medical education

The call for reform of medical curricula in Saudi Arabia took place in 2000, based on current trends in education to meet the requirements of global medical education standards and accreditation.^5^ The goals of these requirements were to emphasize the meaning and understanding of concepts and principles; integrate the teaching of different disciplines in basic sciences, provide early clinical exposure; and enhance clinical skills learning.^6^ In light of these demands, Saudi's medical schools have undertaken significant steps to update their medical education systems.^6^ However, with changes in clinical practice and social demands, there is a need to move towards an innovative and integrated student-centered curricula.^7^ PBL is an educational approach designed to achieve these diverse goals.^8^ In Saudi Arabia, PBL was first adopted by Qassim medical school in 2001, followed by several medical colleges making efforts to explore their own curricula.^6^ UBCOM was established in 2014 with the underlying principle of contributing to  development of health status in Saudi Arabia. The educational program in UBCOM intends to be an integrated, student-centered and incorporate system-based modules that are utilizing hybrid PBL as the primary tool for teaching and learning. This choice came up after an extensive review of current curricula in Saudi Arabia and many parts of the world. The integrated curriculum in UBCOM is a 5-year program spread over three phases of basic medical sciences (Phase I), pre-clerkship (Phase II), and the clerkship (Phase III). These phases begin after the first year, which provides students the important basic sciences principles that form the foundation of subsequent undergraduate medical study.

The courses in Phase I, II and III are presented in modules that vary in their duration from two, to a maximum of ten weeks. Phase I makes up the first cycle of the curriculum which is an introduction to the basis of medical practice regarding basic knowledge of the human body and function in health and disease. These concepts are taught through eight modules presented one after another.  Phase II helps students integrate the knowledge they have acquired during Phase I and prepares them for the subsequent Phase III clerkship. This phase (II) includes eight modules of body organs/systems and six other modules, including Basic Epidemiology, Scientific Research, Clinical Pharmacology, Public Health, Non- communicable Diseases and Clinical Skills.

### Teaching and assessment methods

PBL is the core educational method that extends during phases I and II. During each module in these phases, only one case scenario is introduced per week, and the numbers of PBL scenarios depend on the length of the module. PBL is blended with other teaching and learning methods including lectures, team-based learning (TBL), seminars, self-directed learning, hospital and community field visits, practical and clinical skill sessions. A number of interactive lectures (5 to 7 per week) are also introduced to stimulate understanding of general concepts and to provide an overview of difficult subject materials in conjunction with the PBL scenarios. Seminars and TBL are used as active learning methods to encourage students’ participation and to develop their critical reasoning, problem-solving and interpersonal and teamwork skills.

Assessment is an essential element for forming a judgment about the program quality and extent of students’ achievement and performance in program outcomes.^8^ At UBCOM, formative and summative assessments are designed to be aligned with the PBL curriculum goals. Students self and peer assessment during PBL tutorials, seminar presentations, assignment, and students/tutors feedback constitute formative assessment and are used for tracking our program’s effectiveness. Conversely, final examinations (theory and practical) and continuous assessment represent main parts of summative assessment that used for students grading in each module. The areas of continuous assessment include mid-exam, PBL tutor assessment, TBL readiness assurance tests, portfolio reflection on weekly activities and seminar assessment tests. These have summative values of 40% of final module grade; 8% corresponded to the assessment of students during PBL tutorials. The tutor assessed students’ performance using a scoring rubric for identifying core competencies and essential skills, including attitude and punctuality, preparedness, participation, knowledge, group skills, critical thinking and relevance of resources.  The similar scoring rubric is also used for students self and peer assessment.

### Issues and challenges

Students who joined UBCOM were taught in traditional teacher-directed classrooms during high school and first year that may affect their contribution and performance in the PBL during Phase I. It has been found that the beginning of the PBL process in medical school is the most challenging phase because there were weak group dynamics and no ‘spoon-feeding’ from lecturers.^9^ This emphasizes the importance of orientation and training workshop for the students to cross the bridge from traditional method to innovative PBL approach.

At UBCOM, the first-year courses such as life sciences, medical ethics, and biostatistics were taught in Arabic, not by the English language. Lack of English proficiency affects students’ abilities to engage in optimal discussion, express ideas and communicate effectively during learning activities.^10^ These highlighted the importance of improving students' abilities and proficiency in the English language. In an attempt to bridge the students' transition from the first year to the further phases smoothly, continuous workshops are going on to reform the introductory courses and set a strategy for implementing English as a primary teaching language in the first year. Also, we are planning to develop the English language course to be comprehensive regarding medical terminology, reading, writing, speaking, and comprehension.

Construction of PBL scenarios is a complicated task that requires co-corporation between the teams of educators, subject experts, and staff training groups.^11^ UBCOM generates its own PBL cases for the various courses in Phase I and II. The case scenario follows the seven principles for efficient problem design in PBL as described in the literature.^12^ Faculty experts are tasked with writing the first draft of the case scenarios according to the instructed guideline provided by the department of medical education.  The team includes a subject expert from each discipline from the module with the full guidance of medical education department. Due to the shortage of subject expertise in medical education, only a few faculty members have written the majority of the scenarios used in the college. Therefore, top priority must be given to recruitment of medical education experts as well as designing a PBL training program for faculty which includes a session on scenario writing.

Literature indicates that students’ assessment during PBL tutorials is complicated because there are many dimensions of students achievements need to be evaluated.^8^^,^^13^ However, at UBCOM we observed variability and uneven assessment between groups and tutors, indicating the need for future development of an assessment instrument to reduce the subjectivity of our scoring rubric.

Operating an excellent PBL curriculum requires skilled and well-trained tutors who are committed to this model of curricula.^11^ Although UBCOM is recruiting appropriate academic staff, some of them have limited experiences in PBL or came from traditional medical schools. This limitation highlights the need for the development of extensive training workshops on the PBL process to ensure its successful implementation. Nevertheless, UBCOM has placed a faculty development program to support its academic staff to enhance their teaching, research, and clinical skills. Each week, a two-hourly discussion is held to prepare faculty to meet the challenges of learning and teaching processes to boost the basics of educational planning, instruction methods and students' assessment with particular emphasis on integration. Parts of these sessions focus on PBL with aiming to prepare faculty for their roles as active facilitators. Additionally, new faculty members act as observers during PBL tutorials to be fully oriented with the process before taking facilitator roles.

## Conclusions

Implementation of a PBL curriculum in UBCOM is challenging and requires extensive efforts to achieve the intended educational outcomes. We identify different factors dealing with PBL approach that should be taken into consideration to reach our goals. These include the importance of skilled and experienced tutors for appropriate planning and facilitating of PBL; the need of students’ preparation prior entrance PBL system; Further improvement the criteria of our PBL assessment tool is required to provide equitable measures and avoid assessment variation between students’ groups in PBL. The faculty training programs should be continued for a better quality of our program.

### Conflict of Interest

The authors declare that they have no conflict of interest.
